# ^68^Ga-DOTATATE PET Identifies Residual Myocardial Inflammation and Bone Marrow Activation After Myocardial Infarction

**DOI:** 10.1016/j.jacc.2019.02.052

**Published:** 2019-05-21

**Authors:** Jason M. Tarkin, Claudia Calcagno, Marc R. Dweck, Nicholas R. Evans, Mohammed M. Chowdhury, Deepa Gopalan, David E. Newby, Zahi A. Fayad, Martin R. Bennett, James H.F. Rudd

Myocardial infarction (MI) healing occurs in 2 phases: first an inflammatory phase, where clearance of necrotic debris occurs, followed by a reparative phase characterized by angiogenesis, granulation tissue formation, and attempts to repair the extracellular matrix. While efficient healing relies on coordinated mobilization of monocytes to the damaged myocardium, with resolution of the acute inflammatory response by ∼10 to 14 days, excessive inflammation impairs myocardial salvage and promotes adverse cardiac remodeling.

In ischemic heart failure, pro-inflammatory macrophages persist long after the formation of healed scar in remote and border zones of the infarcted, remodeled heart because of maladaptive changes in the mononuclear phagocytic network and spleen (1). An accurate means of diagnosing harmful inflammation after an MI is urgently needed.

We previously demonstrated that ^68^Ga-DOTATATE, a somatostatin receptor subtype-2 positron emission tomography (PET) ligand, could identify pro-inflammatory macrophages within atherosclerotic plaques (2). Here, in this substudy of our original prospective observational study, we examined whether ^68^Ga-DOTATATE could reveal *residual* post-infarction myocardial inflammation.

Patients with an MI within 3 months treated by percutaneous coronary intervention (“recent MI,” n = 6), and patients with a past history of MI and echocardiography data available from after their event (“old MI,” n = 6), were included. Patients with equivocal culprit arteries, and those managed medically or with coronary artery bypass grafting surgery, were excluded.

ECG-gated PET imaging was performed as previously described (2). Maximum standardized uptake values (SUV_max_) and tissue-to-blood ratios (TBR_max_), normalized for blood pool activity in the superior vena cava, were derived blinded to clinical details in each of the 16 myocardial segments.

Myocardial ^68^Ga-DOTATATE and ^18^F-FDG PET signals were compared: 1) within infarcted and noninfarcted segments; 2) to each other; and 3) to tracer activity in the thoracic vertebral bone marrow as an experimental marker of systemic inflammation, using standard nonparametric statistical tests (all data median [interquartile range (IQR)] unless stated). Recently infarcted myocardial segments were defined by clinically adjudicated (treated) culprit artery territories, with individual anatomical variation verified by angiography. In patients with old MI, infarcted myocardium was determined by echocardiographic wall motion abnormalities (hypokinesia/akinesia), assessed independently of the study and prior to enrollment.

Demographics were similar for recent MI (age 74 years [IQR: 64 to 78 years], 83% male) and old MI (age 59 years [IQR: 56 to 72 years], all male) patients. There were 3 ST-segment elevation MIs, which were all old MIs. PET imaging occurred 35 days (range 21 to 80 days) after recent MIs, and 7 years (range 1.8 to 22 years) after old MIs, with 2 days (range 1 to 21 days) in between ^68^Ga-DOTATATE and ^18^F-FDG scans.

^68^Ga-DOTATATE signals were higher in infarcted compared with noninfarcted myocardium in patients with both recent MI (SUV_max_ 1.60 [IQR: 1.45 to 2.11] vs. 1.33 [IQR: 1.25 to 1.52]; p = 0.03; TBR_max_ 2.33 [IQR: 1.55 to 2.71] vs. 1.80 [IQR: 1.32 to 2.22]; p = 0.03) and old MI (SUV_max_ 2.22 [IQR: 2.03 to 2.50] vs. 1.78 [IQR: 1.63 to 2.13]; p < 0.0001; TBR_max_ 2.79 [IQR: 2.47 to 3.23] vs. 1.89 [IQR: 1.52 to 2.36]; p < 0.0001).

Unlike ^68^Ga-DOTATATE, which exhibited very low background myocardial binding in all patients, avid myocardial ^18^F-FDG uptake (basal inferoseptum SUV_max_ >5) rendered 5 (42%) scans uninterpretable despite 6-h pre-scan fasting. In the readable scans, the 2 tracers showed reasonable agreement in the myocardium (r = 0.38, 95% confidence interval [CI]: 0.20 to 0.53; p < 0.0001). Despite high liver and spleen ^68^Ga-DOTATATE activity, focal myocardial signals were clearly distinguishable in all 5 patients with inferior infarcts.

Bone marrow ^68^Ga-DOTATATE signals were highly correlated with both infarct-related myocardial inflammation detected by ^68^Ga-DOTATATE (r = 0.83 [95% CI: 0.48 to 0.95]; p = 0.001), and metabolic bone marrow activity measured by ^18^F-FDG (r = 0.64 [95% CI: 0.08 to 0.89]; p = 0.03).

**Figure 1:**
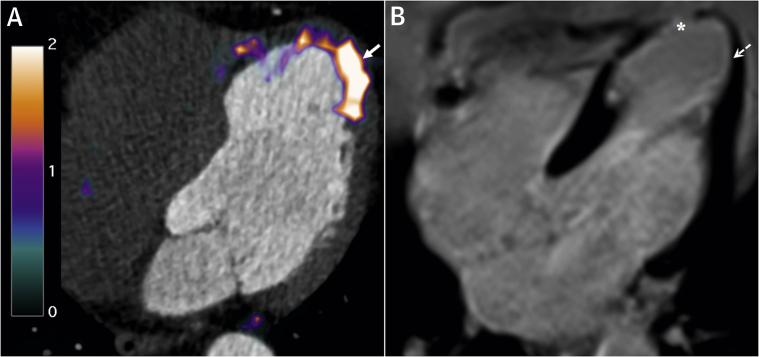
Post-Infarction Myocardial Inflammation Identified by ^68^Ga-DOTATATE PET **(A)**^68^Ga-DOTATATE positron emission tomography (PET)-computed tomography image (scale bar: standardized uptake values) demonstrating residual inflammation **(arrow)** in **(B)** partially viable myocardium with subendocardial infarct **(dashed arrow)**, bordering full-thickness scarring **(asterisk)** confirmed by late gadolinium enhancement magnetic resonance imaging, 4 years after a left anterior descending artery myocardial infarction. ^18^F-FDG positron emission tomography imaging reproduced a near-identical pattern of abnormal myocardial tracer uptake. Stress magnetic resonance imaging was negative for ischemia.

We found that ^68^Ga-DOTATATE identified active inflammation in recently infarcted myocardium, as well as old ischemic injury. Our observations agree with existing clinical data (3), but contradict findings in mice (4). ^68^Ga-DOTATATE binding in chronically damaged myocardium, particularly at the infarct border (Figure 1), likely reflects residual macrophage-driven inflammation; however, histological validation is needed. While tracer binding to myocytes and/or fibroblasts are possible alternative explanations, transcriptomic data from infarcted mouse hearts (5) indicates that *SSTR2* is not expressed in these cell types.

Residual myocardial inflammation detected by ^68^Ga-DOTATATE could represent an important prognostic biomarker to study disease mechanisms and test novel therapies for the inflamed, failing heart.
